# The Effects of Climate Change on *Sthenoteuthis oualaniensis* Habitats in the Northern Indian Ocean

**DOI:** 10.3390/ani15040573

**Published:** 2025-02-17

**Authors:** Lihong Wen, Heng Zhang, Zhou Fang, Xinjun Chen

**Affiliations:** 1 College of Marine living Resource Sciences and Management, Shanghai Ocean University, Shanghai 201306, China; wen009988@outlook.com (L.W.); xjchen@shou.edu.cn (X.C.); 2 College of Oceanography and Ecological Science, Shanghai Ocean University, Shanghai 201306, China; 3 East China Sea Fisheries Research Institute, Chinese Academy of Fisheries Science, Shanghai 200090, China; zhangziqian0601@163.com; 4 National Engineering Research Center for Oceanic Fisheries, Shanghai Ocean University, Shanghai 201306, China; 5 Key Laboratory of Sustainable Exploitation of Oceanic Fisheries Resources, Ministry of Education, Shanghai Ocean University, Shanghai 201306, China; 6 Key Laboratory of Oceanic Fisheries Exploration, Ministry of Agriculture and Rural Affairs, Shanghai 201306, China

**Keywords:** climate change, environmental factors, *Sthenoteuthis oualaniensis*, habitat, northern Indian Ocean

## Abstract

The northern Indian Ocean is located in a typical monsoon region that is also influenced by climate events such as the Indian Ocean Dipole (IOD), which makes *Sthenoteuthis oualaniensis* habitat highly susceptible to changes in climate and marine environmental conditions. This study established a suitability index (SI) model and used the arithmetic average method to construct a comprehensive habitat suitability index (HSI) model based on *S. oualaniensis* production statistics in the northern Indian Ocean from 2017 to 2019. Variations in the suitability of *S. oualaniensis* habitat during different IOD events were then analyzed. The results indicate that the model performed best when year, month, latitude, longitude, sea surface temperature (SST), wind speed (WS), and photosynthetically active radiation (PAR) variables were included in the generalized additive model (GAM). SST, WS, and PAR were identified as the most important key environmental factors. The HSI model showed that the most suitable habitat during a positive IOD event was smaller than during a negative IOD event and that the suitable habitat’s center was located west of the positive IOD event and east of the negative IOD event. There was a significant inverse relationship between the area, suitable for habitation, and the north–south shift in the latitudinal gravity center and the Dipole modal index (DMI). The results indicate significant differences in the habitat of *S. oualaniensis* in the northern Indian Ocean during different IOD events, as well as differences in suitable habitat ranges and the spatial distribution of the species.

## Simple Summary

*Sthenoteuthis oualaniensis* is an economically important species in the northern Indian Ocean, with its suitable habitat being highly sensitive to changes in marine environmental conditions. The northern Indian Ocean is located in a typical tropical monsoon region and is strongly influenced by climate events such as the Indian Ocean Dipole (IOD). The IOD is commonly represented by the Dipole Mode Index (DMI), which is defined as the difference in the average sea surface temperature anomaly between the tropical western Indian Ocean (10° S–10° N, 50° E–70° E) and the tropical eastern Indian Ocean (10° S–0°, 90° E–110° E). This study used a habitat suitability index (HSI) model to analyze the effects of different IOD events on the habitat of *S. oualaniensis*. The results showed that the suitable habitat area was smaller during positive IOD events and larger during negative IOD events. These findings provide valuable scientific insights for developing sustainable management strategies for this species in the context of climate change.

## 1. Introduction

*Sthenoteuthis oualaniensis* is a cephalopod, a member of the family Ommastrephidae. It plays a crucial role in the marine food chain, serving as a significant food source for various marine organisms, including sharks, whales, large fish, and birds; it also acts as a predator, preying on other species. This makes it a key species in marine ecosystems [1,2], and its presence is essential for maintaining species diversity and ensuring the stability of the food web in marine ecosystems. Additionally, *S. oualaniensis* holds significant economic value in the fisheries sector, with the FAO estimating that its total resources are approximately 8–11.2 million tons [3]. *S. oualaniensis* is a globally economically significant oceanic cephalopod with a wide distribution in tropical and subtropical waters of the Pacific and Indian Oceans [4,5]; for example, this squid is a key fisheries target in certain coastal nations bordering the Indian Ocean, as well as in island regions of the western Pacific [6]. It also provides employment opportunities and economic income for local communities. *S. oualaniensis* is one of the most abundant squid species in the northern Indian Ocean; therefore, its resources in this region have important exploitation potential [7].

Most cephalopods have short life cycles, high metabolic rates, fast growth rates, and marked sensitivity to environmental variations; thus, they are associated with high plasticity, which is reflected in the large year-to-year fluctuations in their population abundance [8]. The abundance and spatial distribution of ommastrephid squids are strongly influenced by large-scale climate variations and regional environmental conditions [9,10]. Various anomalous climatic events induce changes in local fishing and spawning grounds of squid species, leading to spatial shifts in breeding and feeding habitats and variability in abundance [11]. For example, Pang et al. [12] found that *Loligo japonica* and *Sepia esculenta* are adaptable to the warmer climate in the North Pacific Ocean, whereas *Sepia esculenta* and *Sepiella maindroni* are not. Yu et al. [13] reported that the abundance of *Todarodes pacificus* exhibited distinct patterns, driven by the marine environment during El Niño–Southern Oscillation (ENSO) periods. Feng et al. [14] concluded that the potential distribution of *Dosidicus gigas* is affected by interannual changes in climate. Chang et al. [15] demonstrated that the Antarctic Oscillation (AAO) induces a two-year lag in the abundance of *Illex argentinus* in the Atlantic Ocean. Liu et al. [16] suggested that changes in Antarctic Sea ice cover will have a significant effect on the temperature of different water layers in the fishing grounds of *Illex argentinus*, thus affecting the distribution of the fish and its resource abundance. Research in the Indian Ocean indicates that there are more *S. oualaniensis* larvae and adults in areas where the positive Indian Ocean dipole (IOD) and El Niño phenomena have strong influences [17]. Thus, changes in climate and marine environments will directly affect the growth and development, reproduction, feeding, and migration patterns of cephalopods, leading to variations in their abundance.

The ENSO is the most potent interannual sea surface temperature (SST) climate anomaly observed in the equatorial Pacific, and its occurrence frequently leads to climate anomalies in many parts of the world, attracting widespread attention [18]. In recent years, a phenomenon akin to El Niño in the adjacent Indian Ocean, referred to as the IOD, has garnered increasing attention from scholars. Saji et al. [19] defined the east–west dipole mode of the tropical Indian Ocean SST on an interannual timescale as the IOD, an event which influences the climate of the Indian Ocean and its adjacent regions (Asia, Oceania, Africa, etc.) through sea–air interactions. The IOD is frequently regarded as the effect of ENSO stress on the tropical Indian Ocean, caused by the ENSO-induced Walker circulation [20,21]. According to some researchers, an IOD event is a self-sustaining phenomenon independent of the ENSO, functioning as a coupled ocean–atmosphere–land system in which the air–sea interaction at the tropical Indian Ocean scale is caused by trade wind anomalies induced by the Mascarene high anomaly in the southern Indian Ocean [19,22]. Until now, the primary conclusion regarding the evolution and mechanism of formation of IOD events has been that they are essentially composed of two distinct modes: one on the scale of the tropical Pacific–Indian Ocean, which is correlated with ENSO, and the other on the scale of the tropical Indian Ocean, which is closely associated with the Indian Ocean local air–sea system [23]. Both modes have similar spatial distributions but exhibit distinct interannual variabilities. Analyses have shown that El Niño events occur during the year of the IOD; however, IOD events do not always occur in conjunction with El Niño phenomena but are more frequently independent events [24,25].

The Indian Ocean Dipole modal index (DMI) is the interannual variation in IOD events and represents the difference in the sea surface temperature anomaly (SSTA) between the tropical western Indian Ocean (10° S–10° N, 50° E–70° E) and the tropical eastern Indian Ocean (10° S–0°, 90° E–110° E) [19]. When the DMI > 0, the IOD is in the positive phase, and the Indian Ocean SSTA is positive in the west and negative in the east; when the DMI < 0, the Indian Ocean Dipole is in the negative phase, and the Indian Ocean SSTA is negative in the west and positive in the east [26]. Previous studies have shown that SST in the eastern Indian Ocean decreases during positive IOD. This is accompanied by changes in thermocline depth and current directions and results in massive phytoplankton blooms and reduced rainfall over adjacent land areas, triggering droughts and forest fires. In contrast, in the western Indian Ocean, SST increases while chlorophyll concentrations decrease, leading to increased precipitation over the African continent and flooding in East Africa. Furthermore, the IOD can influence the climate of Europe, North America, South America, and southern Africa through teleconnection processes [17,27], indicating that IOD has significant impacts on climate at both regional and global scales.

Only a few studies on Indian Ocean fisheries have focused on the IOD and DMI. Jonson et al. [28] found that, during the negative IOD that occurred from 2016 to 2017, the production of small-scale pelagic fish in a fishing port near Java in the eastern Indian Ocean decreased, while production increased during the positive IOD in 2019. *S. oualaniensis*, a significant economic cephalopod in the Indian Ocean, is resource-rich and has enormous development potential [28]. Like other oceanic cephalopods, *S. oualaniensis* habitat is vulnerable to marine environmental and climate change factors. The distribution of its habitat is primarily determined by environmental factors such as SST and climatic events (IOD, ENSO, etc.) [29,30]. However, it is not known how climatic factors influence the marine environment in the Indian Ocean and result in changes in the habitat; for example, how do IOD events affect the offshore marine environment? How does this change affect the abundance of resources and the distribution of the habitat of *S. oualaniensis*? These are critical questions that need to be answered. Using Indian Ocean habitat distribution and IOD data, this study analyzed the differences in the distributions and response mechanisms of Indian Ocean *S. oualaniensis* under various climate models. The results provide a deeper understanding of how climate change impacts this species in the Indian Ocean. Ultimately, the findings offer theoretical support for the restoration of fisheries resources and ecological recovery in the region.

## 2. Materials and Methods

### 2.1. Fisheries, Environmental, and Climatic Index Data

Fisheries data (High Seas Trawling Technology Group, Shanghai, China) collected between 2017 and 2019 were processed into monthly temporal resolutions with a spatial resolution of 0.5° using light casting. The study area encompassed the *S. oualaniensis* fishing grounds in the northern Indian Ocean, roughly bounded between 50° E–75° E and 10° N–30° N. Statistical information included the date, time, fishing location, and catch.

Previous research has established that sea surface temperature (SST), sea surface height (SSH), sea surface salinity (SSS), photosynthetically active radiation (PAR), wind speed (WS), and current speed (U) all play a role in squid survival [30,31]. As a result, this study incorporated environmental factors affecting *S. oualaniensis* fishing grounds. SST and PAR data were obtained from the National Ocean and Atmospheric Administration (NOAA) in the Pacific Ocean Observation Network. Available online: https://oceanwatch.pifsc.noaa.gov/erddap/index.html (accessed on 14 January 2021). SSS, SSH, WS, and U data were obtained from the Asia-Pacific Data Research Center. Available online: https://apdrc.soest.hawaii.edu/ (accessed on 14 January 2021). Prior to habitat model construction, all the environmental data were converted to 0.5° spatial resolutions and monthly resolutions to match the fishery data.

The DMI was used to define IOD events. The DMI data used in this study were derived from publicly available resources at the National Oceanic and Atmospheric Administration (NOAA) Climate Prediction Center website Available online: https://psl.noaa.gov/data/timeseries/month/DS/DMI/ (accessed on 8 December 2021).

### 2.2. Catch per Unit Effort

The catch per unit effort (CPUE) can be used to characterize *S. oualaniensis* resource density. To correspond with the primary operation period of *S. oualaniensis* in the Indian Ocean, data from January to March and from October to December were used to calculate catch and operation times in a 0.5° × 0.5° spatial range; catch per unit of fishing effort was also calculated. The following formula was used for the calculation [32]:

(1) $$\mathrm{CPUE}=\frac{C}{E}$$

where t/time is the unit of the CPUE, *C* is the daily output of a fishing boat, *E* is the corresponding number of operations, light lift and light casting nets are calculated based on the number of nets, and jigging is calculated based on the daily number of changes in the operation position. The resolution was set to 0.5° × 0.5°, and the environmental and fishery data were matched with the fishery data (time, latitude and longitude, catch, and CPUE) using the kriging interpolation method.

### 2.3. Generalized Additive Model

Understanding the relationship between environmental factors and catch is necessary for the rational development and utilization of fishery resources; this relationship is not a simple linear relationship. The generalized additive model (GAM) is a nonlinear relationship model that is a nonparametric extension of the generalized linear model (GLM). It has been extensively used in the study of fishery resources, both domestically and internationally [33,34,35]. In this study, the GAM was used to fit and analyze the abundance of *S. oualaniensis* resources and environmental factors, and those factors with the greatest effect were chosen.

By relocating the function and corresponding variables, the GAM integrates exponential regression with general linear regression [36].

(2) $$\begin{array}{c} \mathrm{In}\;(\mathrm{CPUE}\;+\;1)\sim s\;(\mathrm{year})\;+\;s\;(\mathrm{month})\;+\;s\;(\mathrm{latitude})\;+\;s\;(\mathrm{longitude})\;+\;s\;(\mathrm{SST})\;+\;s\;(\mathrm{SSS})\;+\\ s\;(\mathrm{PAR})+s\;(\mathrm{WS})+s\;(U)+\mathrm{factor}\;(\mathrm{FT})+\varepsilon \end{array}$$

where CPUE + 1 is logarithmically transformed to avoid a zero value in the response variable, *s* is natural cube spline smoothing, *s* (year) is the annual effect, *s* (month) is the monthly effect, *s* (latitude) is the latitudinal effect, *s* (longitude) is the longitudinal effect, *s* (SST) is the sea surface temperature effect, *s* (SSS) is the effect of sea surface salinity, *s* (PAR) is the photosynthetic effective radiation effect, *s* (WS) is the wind speed effect, and *s* (U) is the velocity effect. Since the numerical composition of the operation mode was too small, the operation mode was processed in the form of a factor (FT). The GAM results indicated that SST had the greatest effect on CPUE, followed by WS and PAR.

### 2.4. Habitat Suitability Index Model

In this paper, the SI values for SST, WS, and PAR were calculated using the CPUE from January to March and October to December of 2017 and 2018. This study assumes that the sea area with the greatest distribution of *S. oualaniensis* resources corresponds to the month with the highest unit fishing effort and catch, and the suitability index (SI) value is set to 1. In addition, when the catch per unit fishing effort is 0, the area with the least distribution of *S. oualaniensis* resources is assumed to correspond to the month with the lowest unit fishing effort and catch, and the SI value is set to 0 [37]. The following formula was used to calculate the observed SI values:

(3) $$\mathrm{SI}=\frac{{\mathrm{CPUE}}_{i}}{{\mathrm{CPUE}}_{i,max}}$$

where ${\mathrm{CPUE}}_{i}$ indicates the total fishing effort at a given class interval for each environmental factor and ${\mathrm{CPUE}}_{i,max}$ is the maximum fishing effort at certain class intervals for various environmental factors.

The estimated SI values and the class interval value for each environmental variable were used as the input factors to fit the SI curve using the least-squares method, which was implemented in the Statistical Package for the Social Sciences (SPSS, Version 25.0; IBM Corporation, Armonk, NY, USA). The fitting formula is shown below [38]:

(4) $${\mathrm{SI}}_{X}=\exp[a\times {\left(X-b\right)}^{2}]$$

where *a* and *b* are the model parameters estimated using the least-squares method by reducing the residuals between the observed and predicted SI values to a minimum; SI_X_ is the SI value of each environmental factor; SI values range from 0 to 1; and X is the class interval value of the environmental factors.

The development of a comprehensive HSI model involved the following steps. Equations for SI_SST_, SI_WS,_ and SI_PAR_ were fitted using the aforementioned data and research methods in conjunction with SST, WS, and PAR data for the northern Indian Ocean’s marine environment from 2000 to 2020. The HSI for *S. oualaniensis* in the northern Indian Ocean was calculated using the arithmetic mean model (AMM). The following equation was used to develop the integrated HSI model [39]:

(5) $$\mathrm{HSI}=\frac{1}{3}\left({\mathrm{SI}}_{\mathrm{SST}}+{\mathrm{SI}}_{\mathrm{WS}}+{\mathrm{SI}}_{\mathrm{PAR}}\right)$$

where SI_SST_, SI_WS_, and SI_PAR_ are the predicted SI values for the environmental factors affecting *S. oualaniensis*. The HSI values ranged from 0 to 1, and an HSI ≥ 0.6 was regarded as indicating a suitable habitat [40]. The HSI values for *S. oualaniensis* in the northern Indian Ocean were calculated from January to March and October to December, and the 21-year average HSI value from 2000 to 2020 was used as the habitat climate value for *S. oualaniensis* in the northern Indian Ocean from January to March and October to December. A climate distribution map of *S. oualaniensis* habitat in the northern Indian Ocean was generated, and its spatial distribution characteristics were analyzed from January to March and October to December.

### 2.5. Habitat Suitability Index Differences Under Different Climate Events

The effect of the positive and negative IODs on the habitat of *S. oualaniensis* in the northern Indian Ocean was analyzed. Positive and negative IODs from 2000 to 2020 were analyzed, as were the marine environmental characteristics of the northern Indian Ocean at various stages. The HSI values for *S. oualaniensis* in the northern Indian Ocean were calculated for January to March and October to December during various climate events. A suitability index (SI) ≥ 0.6 for each environmental factor indicated a suitable habitat for *S. oualaniensis*. The spatial distribution of suitable habitats for *S. oualaniensis* in the northern Indian Ocean during various climate event periods was depicted on a map of the study area, as were the impacts of climatic events on *S. oualaniensis*. The relationships between the DMI and changes in suitable habitats for *S. oualaniensis* in different months were analyzed.

## 3. Results

### 3.1. Generalized Additive Model

**Figure 1:**
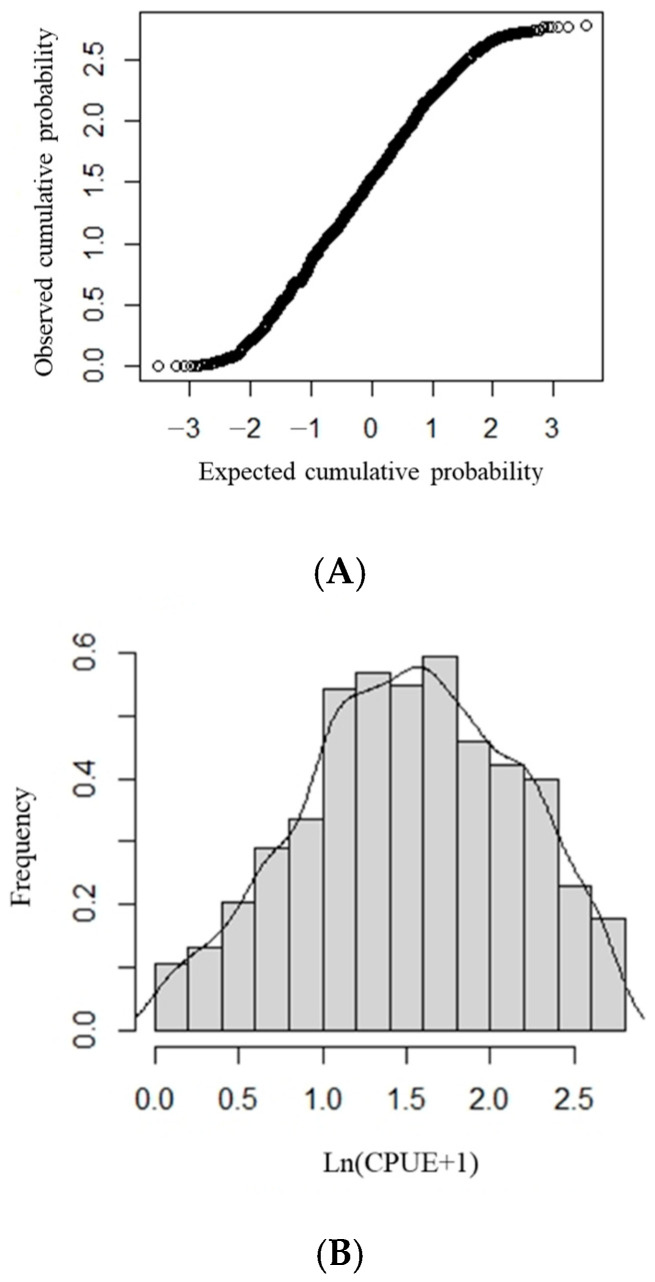
The ln (CPUE + 1) frequency distribution and distribution tests for *Sthenoteuthis oualaniensis* in the northern Indian Ocean. (**A**) Normal Q-Q plot of ln (CPUE + 1). (**B**) Frequency distribution of ln (CPUE + 1).

First, we determined whether ln (CPUE + 1) follows a normal distribution. Using the K-S test, we determined whether the ln (CPUE + 1) datapoints formed a straight line in the normal Q–Q diagram (Figure 1A); if they did, ln (CPUE + 1) followed a normal distribution (Figure 1B). This demonstrates that the assumption that ln (CPUE + 1) follows a normal distribution is reasonable in this study and that the data can be analyzed using the GAM.

The GAM analysis demonstrated that when the explanatory variables—year, month, latitude, longitude, SST, WS, and PAR—were included in the GAM, the Akaike information criterion (AIC) value was smallest and the fitting effect was best (Table 1); thus, these factors were chosen to establish the model.

ln (CPUE + 1)~*s* (Year) + *s* (Month) + *s* (Latitude) + *s* (Longitude) + *s* (SST) + *s* (WS) + *s* (PAR)

The *p*-values indicate that the year, month, latitude, longitude, SST, WS, and PAR significantly affect CPUE (*p* < 0.05). The model explained 21.6% of the total variance in the CPUE (Table 1). Among these variables, the annual variable had the greatest effect on CPUE, accounting for 5.6% of the total deviation, indicating that year has the greatest effect on CPUE. The effects of the month (5.22%), latitude (4.94%), SST (3.94%), longitude (1.2%), WS (0.6%), and PAR (0.1%) were then considered.

### 3.2. Climatic Habitat Distribution

**Figure 2:**
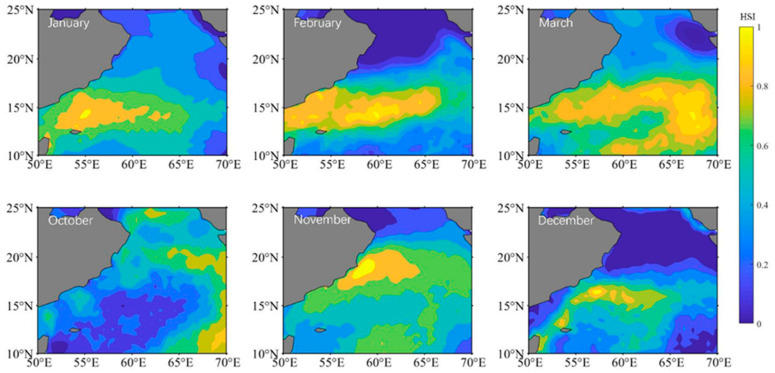
The distribution of HSI climatic area for *Sthenoteuthis oualaniensis* in the northern Indian Ocean from January to March and October to December.

The distribution of *S. oualaniensis* climatic habitat in the northern Indian Ocean was compared from January to March and from October to December (Figure 2). The overall trend in the climate distribution from October to March in the following year was east of 60° E to the west coast of 60° E and then east of 60° E again. Between October and November, the area of the climatic habitat expanded and the center of the suitable habitat shifted to the northwest. The climatic habitat area gradually expanded between December and March of the following year, making it suitable for eastward expansion and the development of the habitat center.

### 3.3. Climate Event Statistics

IOD exhibits phase-locking characteristics. It typically develops in the Northern Hemisphere during the summer, reaches a peak in the autumn, and then subsides during the winter [20,41]. According to Lu et al. [42], a positive (negative) IOD even occurs if the inter-hemispheric pressure gradient (IHPG) index is above (below) the normal temperature from May to August and the DMI is above 0.5 °C (below −0.5 °C) from September to November. This study inferred the abnormal climate events that occurred between 2000 and 2020 based on this definition. Positive IOD events occurred during this time period in 2006, 2012, 2015, 2018, and 2019, while negative IOD events occurred in 2010 and 2016. In 2019, a significant positive IOD event occurred; in 2016, a significant negative IOD event occurred.

### 3.4. Habitat Suitability Index at Various Stages

**Figure 3:**
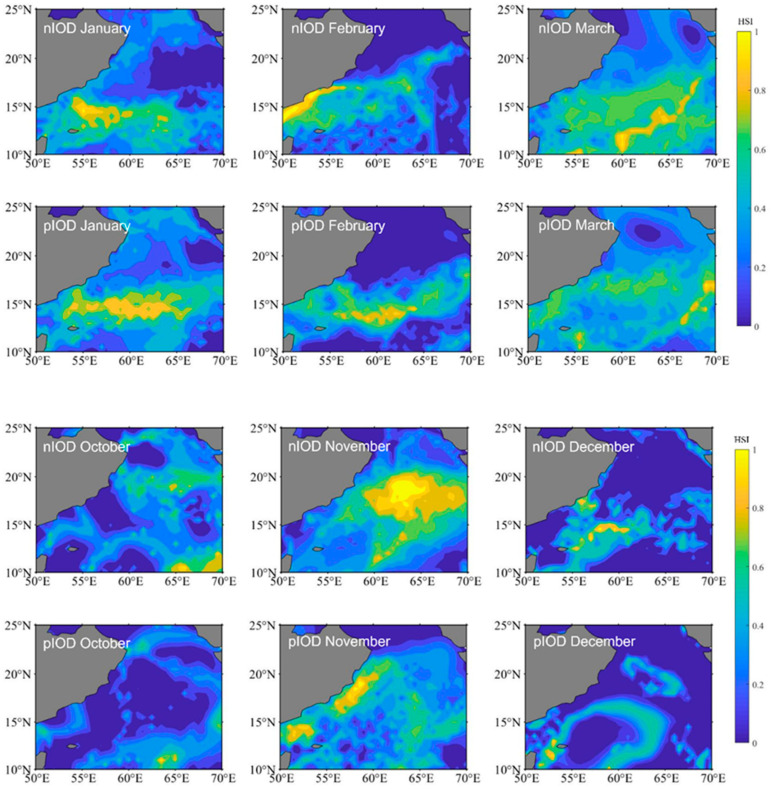
The distribution of *Sthenoteuthis oualaniensis* habitats in the northern Indian Ocean from January to March and October to December during different climatic events. nIOD stands for the negative Indian Ocean Dipole. pIOD stands for the positive Indian Ocean Dipole.

We selected 2016 (a negative IOD event) and 2019 (a positive IOD event) to map the spatial distributions of the HIS for *S. oualaniensis* in the northern Indian Ocean from January to March and October to December, respectively (Figure 3). The study revealed that, between January and March and between October and December, there were differences in suitable habitats for *S. oualaniensis* in the northern Indian Ocean. Specifically, in January and February, the suitable habitat area was greater during positive IOD events compared with negative IOD events, whereas in the remaining months, the suitable habitat area during positive IOD events was smaller than during negative IOD events. The spatial distribution of suitable habitats during different climate events was as follows: the negative IOD event was primarily distributed in the western sea area at 57° E from January to February, whereas the positive IOD event was primarily distributed in the eastern sea area at 57° E from January to February; the negative IOD event had wider latitudinal and longitudinal distributions in March, October, and December compared to the positive IOD event.

### 3.5. Temporal and Spatial Changes in Suitable Habitats

**Figure 4:**
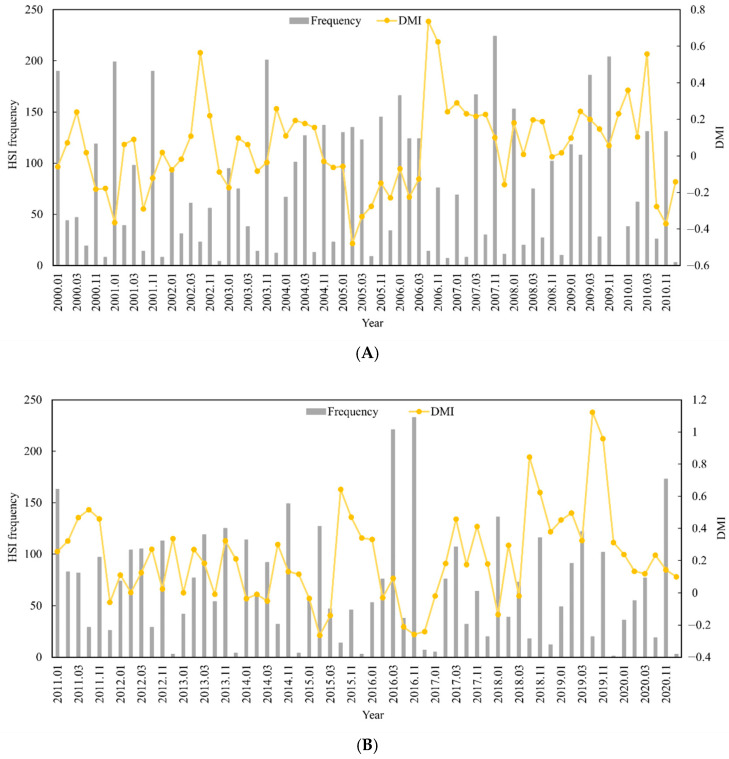
Suitable HSI frequency statistics and monthly DMIs for *S. oualaniensis* in the northern Indian Ocean from January to March and October to December of 2000 to 2010 (**A**). Suitable HSI frequency statistics and monthly DMI variation trends for *S. oualaniensis* from January to March and October to December in the northern Indian Ocean from 2011 to 2020 (**B**).

The relationship between the change in the suitable habitat area and the DMI in different months was analyzed by calculating the suitable habitat area (HSI ≥ 0.6) for *S. oualaniensis* from January to March and October to December in the northern Indian Ocean from 2000 to 2020. The results indicated that, as the DMI increased, the suitable habitat area decreased, and the suitable habitat area was significantly negatively correlated with the DMI (r = −0.197, *p* < 0.05) (Figure 4A,B). In other words, the suitable habitat area for *S. oualaniensis* in the northern Indian Ocean increased from January to March and from October to December, and the opposite trend was detected for the positive IOD event. These findings indicate that climate events had a significant effect on the area suitable for *S. oualaniensis* habitat in the northern Indian Ocean.

**Figure 5:**
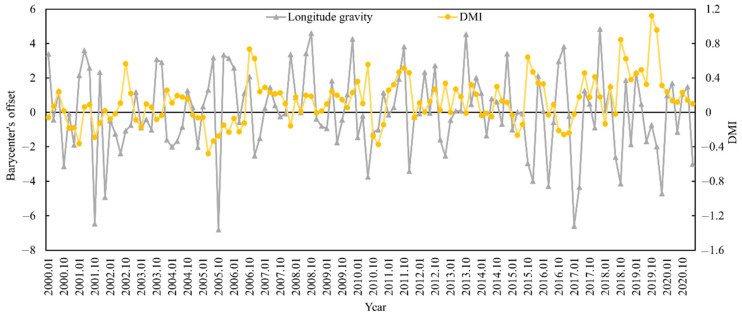
The relationship between HSI longitude center of gravity and annual DMI for *S. oualaniensis* from January to March and October to December in the northern Indian Ocean from 2000 to 2020.

**Figure 6:**
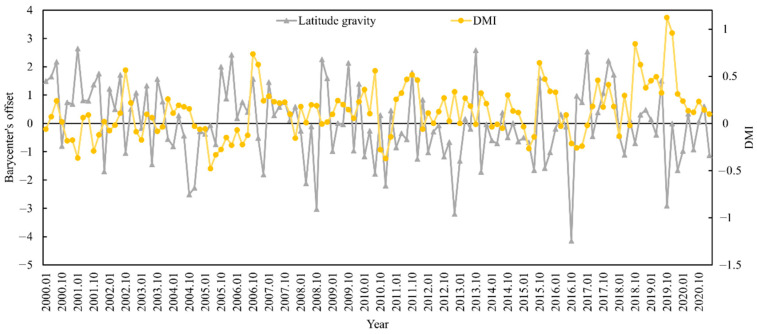
The relationship between HSI latitude center of gravity and annual DMI for *S. oualaniensis* from January to March and October to December in the northern Indian Ocean from 2000 to 2020.

The appropriate habitat (longitude and latitude) for *S. oualaniensis* in the northern Indian Ocean (HSI ≥ 0.6) was determined from January to March and October to December 2000 to 2020. The relationships between the changes in the trends of suitable habitat’s longitude and latitude centers of gravity and the DMI were examined (Figure 5 and Figure 6). Based on correlation analysis, the results indicate that the deviation between the longitudinal and latitudinal barycenters of *S. oualaniensis* in the northern Indian Ocean was inconsistent with the DMI. There was no significant correlation between the east–west shift in the longitude or the center of gravity of *S. oualaniensis* and the DMI (r = −0.114, *p* > 0.05); however, there was a significant negative correlation between the north–south shift in the latitude and the DMI (r = −0.181, *p* < 0.05).

## 4. Discussion

The HSI model is a tool for assessing the quality of fish habitats and has been widely used in recent years in fishery science [43,44]. Based on the results of the GAM and previous studies, this study demonstrated that marine conditions, such as SST, WS, and PAR, have significant effects on the distribution and abundance of cephalopods. Because *S. oualaniensis* is a warm ocean species, the SST directly affects its growth, reproduction, and migration and is one of the primary factors contributing to habitat change [45,46]. The northern Indian Ocean is the world’s most typical monsoon circulation sea area. Monsoons are major drivers of marine environmental change and the dynamic processes of marine ecology in the northern Indian Ocean [47]. A monsoon causes surface seawater to drift with the wind, and deep seawater upwells to the surface so that the deep seawater meets the surface water. The entire marine environment changes as the monsoon climate changes, bringing regional differences and seasonal changes that affect the temporal and spatial distributions of the habitat of *S. oualaniensis* [48]. Additionally, changes in the climate and marine environment affect the spectral components and organic matter–energy in solar radiation, which is useful for plant photosynthesis. These changes affect the quantity and distribution of marine phytoplankton. This, in turn affects, the distribution of *S. oualaniensis* bait organisms, potentially affecting *S. oualaniensis* fishery resources and the temporal and spatial distribution of fishing grounds in the northern Indian Ocean [49,50]. Three critical environmental variables—SST, WS, and PAR—were chosen as the input parameters for the HSI model. The three environmental variables contributed to the construction of the HSI model based on the arithmetic mean method. Because this more accurately reflects the degree of influence of each environmental factor and has been successfully applied to the prediction of suitable habitats for a variety of fish in recent years, the prediction results are more accurate.

### 4.1. Distribution of S. oualaniensis Habitats

The results indicated that, from October to March, the general trend of squid presence in the northern Indian Ocean was east of 60° E to west of 60° E and then back to east of 60° E. Due to the monsoon’s year-round control of the northern Indian Ocean, Kelvin waves are generated as a result of dynamic forcings dominated by wind stress, thermal forcings dominated by sea surface heat flux, and the Coriolis force [51]. Through horizontal propagation, vertical propagation, and eastern boundary reflection, the Kelvin wave modulates the formation and evolution of the equatorial current system in the equatorial mixing layer, subsurface layer, and middle and deep layers; the Kelvin wave propagates to the equator at the basin’s western boundary, while the eastern boundary propagates to the pole. The equatorial Kelvin wave propagates eastward and “strikes” the basin’s eastern boundary, where a portion of the energy is reflected back as a Rossby wave, and the remainder is transmitted to the poles as a coastal Kelvin wave [52]. Because wave energy is transmitted to the outer equator via coastal Kelvin waves and reflected Rossby waves at the eastern boundary, equatorial dynamic processes regulate changes in the circulation structure at the outer equator, as well as marine heat exchange and marine ecological processes in the northern Indian Ocean [53], thereby affecting the distribution of *S. oualaniensis* resources. Between October and November, the monsoon in the equatorial Indian Ocean switches from winter to summer in the Northern Hemisphere. At this time, the equatorial Indian Ocean is controlled by the westerly wind, which stimulates the eastward transmission of equatorial Kelvin waves, causes high temperatures and high-salt water on the western surface eastward, and accumulates on the west bank of Sumatra Island, where it reflects a westward Rossby wave on the eastern boundary. Additionally, the equatorial Kelvin wave affects the upper ocean mass and heat distribution near the equator. In general, *S. oualaniensis* fishing grounds in the Indian Ocean are strongly influenced by temperature, with *S. oualaniensis* tending to migrate toward areas with higher sea surface temperatures [54]; thus, beginning in October, their habitat begins from east of 60° E and expands to the west of 60° E. The northeast monsoon controls the Indian Ocean from December to March of the following year, and there is a westerly northeast monsoon current in the sea area north of 4° N that transports the low-salt water in the Bay of Bengal to the Arabian Sea. At this time, the equatorial Indian Ocean experiences an easterly wind component, which stimulates the equatorial surface current to the west. The current environment exchanges deep and surface seawater, transporting nutrients from deep seawater to the surface, resulting in increased primary productivity, phytoplankton biomass enrichment, and the formation of a favorable habitat environment for fish resources [55]. As a result, the habitat expands east of 60° E again after November. The climate habitat distribution for *S. oualaniensis* in the northern Indian Ocean between January and March and between October and December essentially reflects the habitat distribution of *S. oualaniensis* in different months; the difference in the climate status of the habitat can also reflect differences in suitable environmental conditions for *S. oualaniensis*.

### 4.2. Habitat Effects on S. oualaniensis Under Different Climatic Conditions

*S. oualaniensis* is extremely sensitive to changes in environmental conditions [56,57]. Large-scale changes in the climate significantly affect the marine environment on which cephalopods depend, prompting *S. oualaniensis* populations to respond rapidly by migrating to suitable habitats. Studies have shown that El Niño and La Niña events can affect the distribution of *S. oualaniensis* resources in the South China Sea [58]. This article examines the changes in suitable habitats for *S. oualaniensis* in the northern Indian Ocean from January to March and October to December 2016 and 2019. The spatial distribution and variation trends for *S. oualaniensis* in the northern Indian Ocean vary based on climate events. Between January and February, there were more positive IOD events than negative IOD events in the suitable habitat area. This could be because the IOD exhibits phase-locking characteristics, which typically start in the Northern Hemisphere in spring, develop during summer, reach the peak phase in autumn, and then dissipate in winter [59]. Therefore, the influence of IOD events is not significant in January and February. Between January and February 2016, the sea surface temperature increased due to the strong El Niño event in 2015, expanding the range of suitable habitats [60]. Between October and December and March, the number of positive IOD events in suitable habitat areas was lower than the number of negative IOD events, and negative IOD events occurred over a wider range of longitudes. Monsoons strengthened during the positive IOD event, resulting in increased coastal upwelling and decreased SST, while the eastern Indian Ocean SST was abnormally cold.

The above results indicate that changes in climate events affect the sea surface of suitable *S. oualaniensis* habitats in the northern Indian Ocean and that changes in the marine environment in the habitat sea area affect cephalopod habitats, which subsequently affects the spatial distribution of suitable habitats. Climate events that occur over time cause changes in the Indian Ocean’s marine environment. The appropriate habitat for cephalopods in the sea area responds to this, and the spatial distribution of habitats varies from year to year.

### 4.3. Spatial Distribution and Regularity of Habitats

The spatial distribution of *S. oualaniensis* habitats with HSI ≥ 0.6 in the northern Indian Ocean from January to March and from October to December demonstrated that the size of the habitat sea area for *S. oualaniensis* varied during different climatic events. The suitable habitat area for *S. oualaniensis* in the northern Indian Ocean generally decreased as the DMI increased, and the suitable habitat area was significantly negatively correlated with the DMI; that is, during the negative IOD event, the area suitable for *S. oualaniensis* in the northern Indian Ocean increased, whereas during the positive IOD event, the area suitable for *S. oualaniensis* decreased. The reason for these results is that, during the positive IOD event, the significantly enhanced equatorial easterly anomaly and the southeast wind anomaly along the coast of Sumatra Island increased upwelling in the southeast Indian Ocean, resulting in thermocline uplift. This increased the zonal thermocline gradient in the equatorial Indian Ocean, increased the pressure gradient force to the east, and subsequently strengthened the equatorial undercurrent. The enhanced equatorial undercurrent continued to transport subsurface water to the upwelling area of the tropical southeast Indian Ocean [42]. This compensates for the upwelling of the equatorial eastern Indian Ocean and contributes to further cooling of the sea surface temperature of the tropical southeast Indian Ocean. The intensification of atmospheric ocean convective activity, cloudy, and rainy weather, and the decrease in photosynthetic effective radiation, all work simultaneously against marine phytoplankton production and reproduction [61]. During the negative IOD event, the westerly anomaly deepens the thermocline of the Indian Ocean, reduces wind speed, weakens sea surface evaporation, reduces ocean latent heat loss, and increases the sea surface water temperature. Additionally, during westerly winds, the atmosphere is in a downdraft, with sunny days and increased photosynthetic effective radiation, conditions conducive for marine phytoplankton accumulation [60,62].

Research findings on the relationships between the trends in the change of longitudinal gravity centers and latitudinal gravity centers and DMI in the suitable habitat of *S. oualaniensis* in the northern Indian Ocean (HSI ≥ 0.6) from January to March and October to December 2000 to 2020 indicate that the east–west shift in the longitudinal gravity center of *S. oualaniensis* has no significant association with the DMI, whereas the north–south shift in the latitudinal gravity center has a significant negative correlation with the DMI. This is due to the meridional heat transport (MHT) that occurs between the atmosphere and the ocean. Meridional heat transport is a vital component of ocean heat redistribution because it connects tropical and subtropical oceans [63]. As a result, the climate variability of marine MHT affects heat distribution in the upper ocean, thus affecting the global climate. The Indian Ocean’s monsoon and IOD are inextricably linked to the climate variability of the marine MHT. Dynamic forcing is dominated by wind stress in the northern Indian Ocean, while thermal forcing is dominated by sea surface heat flux. The northern Indian Ocean receives heat annually, and the ocean heat is transported southward across the equator via meridional flow [64]. The meridional heat transport in the Indian Ocean is dominated by the Ekman region of the upper ocean and is associated with the net heat flux at the sea surface and the meridional cross-equatorial current [65].

## 5. Conclusions

In this study, the significant effects of environmental factors on the CPUE of *S. oualaniensis* in the northern Indian Ocean were examined. By employing a well-established and widely used HSI model, the habitat suitability index for *S. oualaniensis* in the region was calculated, and the influence of IOD events on suitable habitats was analyzed under large-scale climate change. The results demonstrate that the total area of suitable habitats is larger during the negative IOD event than during the positive IOD event. Additionally, the center of suitable habitats shifts toward the western region during the positive IOD event and moves eastward during the negative IOD event. Based on projected changes in environmental factors and forecasts of climate events, it is recommended that the spatiotemporal dynamics of suitable *S. oualaniensis* habitats should be simulated and appropriate fishing and conservation strategies should be developed, thereby ensuring the healthy and sustainable development of this resource.

## Figures and Tables

**Table 1:** Statistical results of the generalized additive model (GAM).

Explanatory Variable	*DF*	*F*	*p*	*R* ^2^	AIC	Cumulative Interpretation Deviation
Null	-	-	-	-	-	-
+year	1.957	69.270	<0.01	0.052	5439.061	5.6%
+month	3.713	28.550	<0.01	0.090	5336.457	10.82%
+latitude	3.485	37.540	<0.01	0.138	5199.324	15.76%
+longitude	2.419	11.820	<0.01	0.149	5166.385	16.96%
+SST	1.856	6.005	<0.01	0.150	5165.758	20.9%
+WS	2.559	7.270	<0.01	0.155	5152.522	21.5%
+PAR	-	-	<0.01	0.210	4979.165	21.6%

*DF*—degree of freedom. *F*—the Fisher test. *p*—corresponding *p*-values. *R*^2^—degree of fitting. AIC—Akaike information criterion.

## Data Availability

The dataset is available on request from the authors.
